# The Prevalence and Associated Factors of Post-COVID-19 Fatigue: A Systematic Review and Meta-Analysis

**DOI:** 10.7759/cureus.63656

**Published:** 2024-07-02

**Authors:** Wangjuan Hu, Rongzhu Tang, Siyuan Gong, Jihong Liu, Jia Li, Chunlian Liao

**Affiliations:** 1 Department of Neurology, The Second Affiliated Hospital of Chongqing Medical University, Chongqing, CHN

**Keywords:** meta-analysis, systematic review, prevalence, post-covid-19 fatigue, covid-19

## Abstract

After the coronavirus disease 2019 (COVID-19) pandemic, numerous individuals experienced the enduring consequences of infection. One of the psychological symptoms that patients report most frequently is persistent fatigue, which is also called post-COVID-19 fatigue. This persistent fatigue can prolong recovery time for hospitalized patients and reduce exercise motivation for residents, affecting their health and working conditions. To determine the prevalence and associated factors, we conducted searches in PubMed, Embase, Web of Science, and Cochrane Library, from inception to 27 March 2023, and a total of 38 studies and 17,738 patients were included in this analysis. We analyzed data and estimated publication bias by Egger’s test and funnel plot by STATA 14. We summarized the prevalence of post-COVID-19 fatigue and calculated the pooled OR to determine associated factors. This study revealed that the prevalence of fatigue in post-COVID-19 syndrome was 46.6% (95% CI: 38.5%-54.7%). Being female (OR: 0.40, 95% CI: 0.24-0.56), older age (OR: 0.04, 95% CI: 0.01-0.07), clinical severity (OR: 0.66, 95% CI: 0.24-1.09), the number of acute COVID symptoms (OR: 3.23, 95% CI: 1.83-5.69), preexisting hypertension (OR: 1.24, 95% CI: 1.08-1.42), lung disease (OR: 2.71, 95% CI: 1.07-6.89), and depression (OR: 1.55, 95% CI: 1.01-2.39) were risk factors for post-COVID-19 fatigue. By revealing the association of these factors with fatigue, it can help us to identify and treat post-COVID-19 fatigue early.

## Introduction and background

During the onset of the coronavirus disease 2019 (COVID-19) pandemic, more than 65 million people have experienced chronic consequences, collectively known as post-COVID-19 syndrome [1,2]. One definition of post-COVID-19 syndrome is a reduction in one's ability to function physically and/or mentally as a result of multiple disease-related factors [3]. According to the National Institute for Health and Care Excellence (NICE), post-COVID-19 syndrome is described as symptoms that develop or remain and last longer than 12 weeks following diagnosis [4]. There are at least 200 symptoms associated with the post-COVID-19 syndrome, including arthralgia, lack of taste and smell, exhaustion, dyspnea, and cognitive decline [5]. The presence of these symptoms results in varying degrees of functional disability, limiting day-to-day activities [6]. It was found that the degree of systemic inflammation during acute COVID-19 infection is intricately connected with the emergence of psychiatric symptoms after viral clearance and that this has a significant impact on survivors' living lives [7].

The post-COVID-19 fatigue belongs to the neuropsychiatric symptom after infection of COVID-19 [8,9]. It appears not only in patients suffering from critical conditions but also in asymptomatic or mildly symptomatic patients [10]. Since it is a common symptom, it is important to clarify its mechanisms, associated factors, mitigation measures, and treatments. Several studies have revealed its pathogenesis and the additional complications it may cause. Some studies have reported similar findings that myopathies may contribute to the occurrence of fatigue, such as myofibrillar degeneration and muscle tissue lesions [11]. However, there is still no definite pathologic mechanism to explain the occurrence of post-COVID-19 fatigue.

Moreover, there is potential for setbacks and functional deterioration if patients suffer from excessive post-COVID-19 fatigue. Many experts have pointed out that the occurrence of fatigue symptoms often leads to a reduction in physical performance, and a decrease in quality of life, and may even cause a series of complications. Multiple studies [12,13] have verified that post-COVID-19 fatigue is likely to result in late-onset pain, decreased exercise capacity, insomnia, and post-exertional malaise [14]. We should give post-COVID-19 fatigue enough attention because it may be detrimental to people's health and quality of life [15].

Post-COVID-19 fatigue is the most prevalent symptom according to an increasing number of studies, and it may be strongly associated with both physiological conditions and demographic traits. Much research has indicated that women are at a higher risk of experiencing post-COVID-19 fatigue. According to a meta-analysis, older survivors and female COVID-19 patients with cerebrovascular illness were more likely to experience fatigue [8]. Another study found that a number of characteristics were linked to the fatigue of infected patients: education, prior chronic illness, dyspnea, and confusion [16]. However, a study in Poland reported that the best scores for fatigue were obtained for subjects with mild COVID-19. Given that numerous studies have reported on the presence and risk factors of post-COVID-19 fatigue, there are some inconsistent opinions. Thus, there is a need to further explore the factors that influence post-COVID-19 fatigue.

Furthermore, current studies have tried many measures to relieve fatigue in people suffering from post-COVID-19 syndrome. That notwithstanding, they have focused on treating post-COVID-19 fatigue through physical therapy rehabilitation, including general physical exercise, supervised exercise programs, rehabilitation robots, aerobic training, or respiratory muscle training [17-19]. However, few of these interventions have targeted specific risk factors. Preventive therapies addressing the reasons for post-COVID-19 tiredness have not received much attention in studies.

Consequently, the primary purpose of this research was to conduct a comprehensive analysis to identify the prevalence of post-COVID-19 fatigue. In addition, we aimed to explore the factors associated with fatigue and to help identify groups at high risk of fatigue to reduce the occurrence of this symptom by intervening in the factors associated with fatigue and reducing the severity of fatigue. Additionally, our results may serve as a foundation for developing clinical risk prediction models for COVID-19-fatigued patients.

## Review

Materials and methods

We performed our literature review using the Preferred Reporting Items for Systematic Reviews and Meta-Analyses (PRISMA) as a guide [20]. The PROSPERO database contains a prospective registration for this investigation (CRD42023416527).

Data Source and Research

The literature research was performed in PubMed, Web of Science, Embase, and The Cochrane Library from inception to 27 March 2023. A combination of the following keywords made up the research strategy: ("COVID-19" OR "SARS-CoV-2" OR "coronavirus" OR "2019-nCoV") AND ("post-acute COVID -19 syndrome" OR "Long COVID" OR "PASC" OR "post-COVID-19 condition" OR "long term" OR "long haul*" OR "after recovery" OR "prolong*" OR "persist*" OR "convalescent") AND ("fatigue" OR "weariness" OR "asthenia" OR "lassitude" OR "exhaustion") (Table 1). We also manually searched relevant studies using the references of the included studies for additional studies. Based on predetermined criteria, two reviewers (W.H. and R.T.) chose studies that met the eligibility requirements. The first step involved removing duplicate studies using EndNote software (Clarivate, Philadelphia). After removing studies that didn't meet the inclusion criteria, the titles and abstracts of the remaining articles were evaluated, and the full texts of those studies were collected. Any discrepancies between the two authors were discussed and resolved by a third reviewer (L.C.)

**Table 1:** The search terms used in PubMed

	Search Terms	Results
#1	"post-acute COVID-19 syndrome"[MeSH Terms]	2,068
#2	"long COVID"[Title/Abstract] OR "PASC"[Title/Abstract] OR "post-COVID-19 condition"[Title/Abstract] OR "long term"[Title/Abstract] OR "long haul*"[Title/Abstract] OR "after recovery"[Title/Abstract] OR "prolong*"[Title/Abstract] OR "persist*"[Title/Abstract] OR "convalescent"[Title/Abstract]	1,886,613
#3	#1 OR #2	1,886,826
#4	"COVID-19"[MeSH Terms]	228,180
#5	"SARS-CoV-2"[Title/Abstract] OR "coronavirus"[Title/Abstract] OR "2019-nCoV"[Title/Abstract]	191,879
#6	#4 OR #5	306,485
#7	"fatigue"[Title/Abstract] OR "weariness"[Title/Abstract] OR "asthenia"[Title/Abstract] OR "lassitude"[Title/Abstract] OR "exhaustion"[Title/Abstract]	153,708
#8	#3 AND #6 AND #7	1,841

Inclusion and Exclusion Criteria

To ensure that the included studies met our research objectives, the inclusion criteria that we developed are as follows: (i) Population: the patients lack a diagnosis of any other illness and have experienced chronic fatigue that began during or after a COVID-19 infection and has persisted for at least 12 weeks [4]; (ii) Intervention: a survey of the patient's symptoms of fatigue was conducted by questionnaire or other methods; (iii) Outcome: studies reporting the prevalence and associated factors of post-COVID-19 fatigue; (iv) Study design: cohort studies, case-control studies, and cross-sectional studies. The following were the exclusion criteria: (i) The language of studies is not English; (ii) The studies are case reports, reviews, meta-analyses, or conference abstracts; (iii) There is no available data or access for full text; (iv) If there are overlapping populations in studies, we prefer to choose the most complete queue.

Study Selection and Data Extraction

Based on predetermined criteria, two authors carried out the screening process to find eligible studies. In the first step, we removed duplicate studies using EndNote software. Then, after eliminating irrelevant studies from the remaining studies' titles and abstracts, the entire texts of the chosen research were perused to determine which ones fulfilled the requirements. Any discrepancy between the two authors was discussed and settled by a third investigator. Two reviewers independently extracted the available data for the final retained studies, and the extracted items included study type, author, publication year, country, total sample size, fatigue sample size, evaluation methods, tools for fatigue assessment, and associated factors. If a discrepancy arose in the process of checking the data, it was necessary to discuss it with the third reviewer to settle the discrepancy.

Quality Assessment

Additionally, the quality of the included studies was evaluated by two authors based on specific scales depending on the study design. For cross-sectional research, we utilized the Agency for Healthcare Research and Quality (AHRQ) scale, and for cohort or case-control studies, we used the Newcastle-Ottawa Scale (NOS) [21]. Each study was evaluated separately by two reviewers, and the outcomes were cross-checked. Any disagreements were settled through conversation with the third author. 

Statistical Analysis

The data extracted to analyze is the odds ratio (OR). In this study, the OR of the associated factors for the occurrence of fatigue was extracted, and finally the OR of each effector was combined, and the pooled OR was used to represent the relationship of the factors associated with fatigue. A meta-analysis was conducted when at least two studies reported risk factors related to post-COVID-19 fatigue, and there were available data used to calculate pooled OR. By the quantitative analysis method, it was extracted directly if the OR was reported in the study. If the study's sample size of individuals who were and weren't fatigued, it was converted to an OR by the formula to calculate the effect sizes at the end. The I^2^ value was used to measure the heterogeneity of the study, and values of 25%, 50%, and 75% indicated low, moderate, and high heterogeneity, respectively [22]. We calculated the pooled prevalence of post-COVID-19 fatigue and the meta-analyses using the random-effects model by the DerSimonian-Laird approach due to the high degree of heterogeneity in the included trials. Sensitivity analyses were conducted on the primary data of the included studies, and subgroup analyses were performed based on geographic location, evaluation methods, and type of studies. To evaluate the publication bias of meta-analysis, the funnel plot and Egger's test were employed [23]. For the robustness of the overall findings, we used a sensitivity analysis to exclude one literature study in turn, and the remaining literature was merged in meta-analysis to assess the robustness and reliability of the merged results of the meta-analysis by observing the changes in the merged results. Statistical significance was determined using a threshold of p < 0.05, and there was a 95% confidence interval (CI) used.

Results

Search Result and Study Characteristics

**Figure 1:**
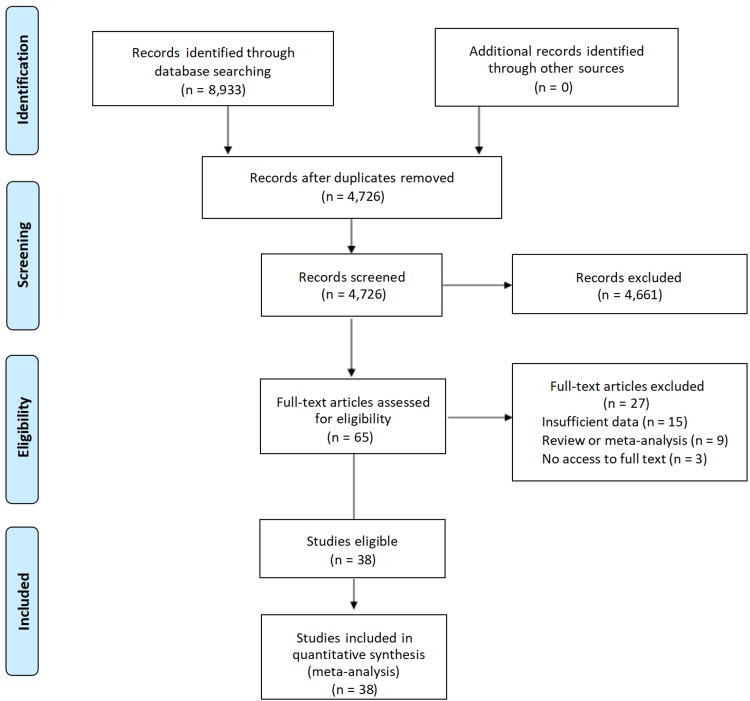
PRISMA flow diagram of the literature research process and results. PRISMA: Preferred Reporting Items for Systematic Reviews and Meta-Analyses

As shown in Figure 1, a total of 8,933 records were identified through the search process. There were 4,207 duplicate studies, and 4,726 studies remained. Then 4,661 records were eliminated after the preliminary screening of the titles and abstracts of the records. Subsequently, the full texts of 65 papers were reviewed. Among these articles, nine studies were reviews or meta-analyses. Additionally, 15 studies did not have sufficient data, and three studies had no access to the full text. Ultimately, the analysis included 38 eligible studies in total.

In this literature review and meta-analysis, we included 38 studies [12,24-60], and the sample sizes ranged from 40 to 2,649 comprising 17,738 cases of COVID-19 infection. The prevalence of fatigue ranged from 3.0% to 90.5%. There were 31 studies belonging to cohort studies, and seven studies were cross-sectional studies. The published time was between 2020 and 2023. According to the countries of 38 studies and the continent divided by the World Health Organization (WHO), eight studies were conducted in Asia, 24 in Europe, two in Africa, and four in America. Among these studies, 38 reported the existence of post-COVID-19 fatigue, and all of them reported the associated factors. Among these included studies, a total of 20 used standardized questionnaires or scales which have been widely used throughout the world, so we categorized these types of evaluation tools as standardized questionnaires or scales. While 10 studies used evaluation tools developed by themselves, such as the self-report from patients, so we categorized these types of tools as simple checklists and interviews. Eight studies did not report their evaluation tools clearly. The majority of the studies discussed factors associated with fatigue, and the comprehensive details about the selected studies are shown in Table 2.

**Table 2:** Characteristics of the included studies. NA: Not available; FSS: Fatigue Severity Scale; CFQ: Chalder Fatigue Scale; FAS: Fatigue Assessment Scale; VAS: Visual Analogue Scale; CIS8R: Checklist of Individual Strength 8R; EQ-5D-5L: EuroQoL five-dimension five-level; CFS: Chronic Fatigability Syndrome Questionnaire; 1: gender; 2: anxiety; 3: depression; 4: VAS score; 5: occupation status; 6: low physical activity; 7: worst cognitive performance; 8: higher ferritin serum levels; 9: BMI; 10: lung disease; 11: comorbidities; 12: age; 13: length of hospital stay days; 14: hypertension; 15: clinical severity; 16: former smoking; 17: current smoking; 18: education; 19: marital status: 20: number of medications; 21: oxygen therapy; 22: number of acute COVID symptoms

Study (Year)	Country	Age	Follow-up	Evaluation tools	Type of evaluation tools	Fatigue cases	Sample size	Fatigue rate (%)	No. of Factors
Adar et al. (2023) [12]	Turkey	53.45±12.76	3 months	FSS	Standardized questionnaires or scales	63	100	63.0	1, 2, 3, 4
Alotibi et al. (2023) [24]	Saudi Arabia	NA	2 months	CFQ	Standardized questionnaires or scales	191	343	55.7	1, 5
Aly et al. (2021) [25]	Egypt	73.18 ± 6.42	1 month	NA	NA	66	98	57.4	NA
Delgado-Alonso et al. (2022) [26]	Spain	46.31 ± 7.97	20.71 ± 6.50 months	NA	NA	24	77	31.2	5, 7
Diem et al. (2022) [27]	Switzerland	Median (IQR) 44.8 (40.4-49.3)	28.0 weeks	FSS	Standardized questionnaires or scales	38	42	90.5	1, 8, 9, 13
El Sayed et al. (2021) [28]	Egypt	36.58 ± 9.85	19 days	FAS	Standardized questionnaires or scales	17	60	28.3	1, 11, 15
Elbéji et al. (2022) [29]	Luxembourg	40 ±13	2 weeks	NA	NA	92	296	31.1	1
Eloy et al. (2021) [30]	France	61	6 months	NA	NA	151	324	46.6	13
Fernández-de-Las-Peñas et al. (2021) [31]	Spain	62	12 months	NA	NA	300	412	72.8	1, 9, 10
Fernández-de-Las-Peñas et al. (2022) [32]	Spain	61 ± 16	8.4 months	NA	NA	1206	1,969	61.3	1, 6
Gil et al. (2023) [33]	Brazil	59 ± 14	6 months	NA	A structured questionnaire applied via a phone call	22	80	27.5	NA
González-Hermosillo et al. (2021) [34]	Mexico	51.0 ± 14	6 months	NA	NA	61	130	46.9	1, 2, 3, 4, 9, 10, 12, 13, 14, 16
Gottlieb et al. (2023) [35]	United States	NA	3 months	The CDC Short Symptoms Screener	Simple checklists and interview	302	2,373	12.7	NA
Grover et al. (2021) [36]	India	36.08 ± 13.12	4–6 weeks	FSS	Standardized questionnaires or scales	126	206	61.2	2, 3, 7
Hartung et al. (2022) [37]	Germany	NA	9 months	The 13-item FACIT fatigue scale	Standardized questionnaires or scales	188	969	19.4	1, 2, 3, 12, 15, 22
Jacobs et al. (2022) [38]	United States	57	35 ± 5 days	PROMIS® survey questions	Simple checklists and interview	104	183	56.8	1, 12
Khatib et al. (2022) [39]	United States	NA	8 months	FAS	Standardized questionnaires or scales	68	157	43.3	1, 5, 9, 16, 19
König et al. (2023) [40]	Netherlands	47.3 ± 12.9	15 months	CIS8R	Simple checklists and interview	150	430	34.9	1, 9, 16, 18, 19, 20, 22
Ladlow et al. (2023) [41]	United Kingdom	NA	159 ± 7 days (5 mo)	FAS	Standardized questionnaires or scales	51	88	58.0	15
Manning et al. (2022) [42]	United States	38.26 ± 12.15)	39 days	Brief Fatigue Inventory	Simple checklists and interview	17	563	3.31	2, 3
Mazurkiewicz et al. (2023) [43]	Poland	30–48	12 weeks	Three steps illustrated in figures	Simple checklists and interview	268	303	88.5	1
Mirfazeli et al. (2022) [44]	Iran	50	9 months	A comprehensive checklist	Simple checklists and interview	49	95	51.0	1, 15
Munblit et al. (2021) [45]	Russia	56	218 days	EQ‐5D‐5L	Standardized questionnaires or scales	562	2,649	21.2	1, 9, 10, 14
Oliveira et al. (2022) [46]	Brazil	Median (IQR) 31.0 (26–36)	13 months	Four types of custom-built questionnaires	Simple checklists and interview	162	588	27.5	15
Sabaner et al. (2022) [47]	Turkey	32.59	NA	FSS	Standardized questionnaires or scales	98	225	43.6	NA
Schouborg et al. (2022) [48]	Denmark	64 + 15	9.5 ± 15.2 days	FAS	Standardized questionnaires or scales	34	80	42.5	1, 4, 10
Sigfrid et al. (2021) [49]	United Kingdom	59·7	222 days	VAS	Simple checklists and interview	271	327	82.9	1, 10, 11, 15
Skinner et al. (2023) [50]	United States	51.1 ± 11.91)	553 days	CFQ-11	Standardized questionnaires or scales	38	48	79.2	13
Sperling et al. (2022) [51]	Denmark	59.9	127.7 days	FAS	Standardized questionnaires or scales	133	218	61.0	2, 3, 21
Stavem et al. (2021) [52]	Norway	Median (IQR) 49.6 (17.7 to 87.9)	4 months	CFQ-11	Standardized questionnaires or scales	211	458	46.1	1, 3, 9, 11, 13, 15, 16, 19, 22
Sun et al. (2022) [53]	China	62	15 months	EQ-5D-5L	Standardized questionnaires or scales	92	534	17.2	1, 11, 13, 21
Thiele et al. (2022) [54]	Germany	61 ± 2	194 ± 3 days	A standardized clinical interview	Standardized questionnaires or scales	17	60	28.3	1, 2, 9, 11, 15, 16, 17
Tleyjeh et al. (2022) [55]	Saudi Arabia	NA	122 days	CFS	Standardized questionnaires or scales	59	222	26.6	1, 9, 10
Townsend et al. (2021) [56]	Ireland	44.5	166.5 days	CFQ-11	Standardized questionnaires or scales	13	40	32.5	1, 2, 5, 9, 11, 20
Twomey et al. (2022) [57]	Canada	NA	3 months	FACIT-F	Standardized questionnaires or scales	152	213	71.4	9
Verveen et al. (2022) [58]	Netherlands	NA	12 months	The validated short fatigue questionnaire	Standardized questionnaires or scales	254	303	83.8	1, 3, 9, 10, 11, 15
Wensink et al. (2023) [59]	Netherlands	59.7 ± 7.65	3 months	21 questions concerning momentary complaints and affects	Simple checklists and interview	11	42	26.2	5
Zhang et al. (2021) [60]	China	Median (IQR) 60.0 (49.0-68.0)	12 months	Self-reported symptom questionnaire	Simple checklists and interview	696	2,433	27.7	1, 12, 14, 15, 16, 17, 21

Quality Assessment

The literature quality was assessed by using the NOS or AHRQ, and the detailed quality assessment scores for studies included can be found in Table 3 and Table 4.

**Table 3:** Quality assessment based on the NOS. NOS: Newcastle-Ottawa Scale

Study	Selection				Comparability		Outcome			NOS score
Adar et al. (2023) [12]	1	1	1	0	1	0	1	0	0	5
Diem et al. (2022) [27]	1	1	0	1	1	0	1	1	0	6
Elbéji et al. (2022) [29]	0	1	1	0	0	0	1	0	1	4
Eloy et al. (2021) [30]	1	1	1	1	1	1	1	1	0	8
Fernández-de-Las-Peñas et al. (2021) [31]	1	0	1	1	1	0	1	1	0	6
Fernández-de-Las-Peñas et al. (2022) [32]	1	0	1	1	1	0	1	1	0	6
Gil et al. (2023) [33]	1	1	1	1	1	0	1	1	0	7
González et al. (2021) [34]	1	1	1	1	1	0	1	1	0	7
Gottlieb et al. (2023) [35]	1	1	1	1	1	1	1	1	0	8
Hartung et al. (2022) [37]	1	1	1	1	1	1	1	1	1	9
Jacobs et al. (2022) [38]	1	0	1	0	1	0	1	1	1	6
König et al. (2023) [40]	1	1	1	1	1	0	1	1	0	7
Ladlow et al. (2023) [41]	0	1	1	1	1	1	1	1	1	8
Manning et al. (2022) [42]	0	1	0	0	1	0	1	1	1	5
Mazurkiewicz et al. (2023) [43]	1	0	1	1	1	0	1	1	1	7
Mirfazeli et al. (2022) [44]	1	0	1	1	1	0	1	1	1	7
Munblit et al. (2021) [45]	1	0	1	1	1	0	1	1	1	7
Oliveira et al. (2022) [46]	1	1	1	1	1	0	1	1	0	7
Sabaner et al. (2022) [47]	1	0	1	1	1	0	1	1	1	7
Schouborg et al. (2022) [48]	1	0	1	1	1	0	1	1	0	6
Sigfrid et al. (2023) [49]	1	0	1	1	1	0	1	1	0	6
Skinner et al. (2023) [50]	0	0	1	1	1	0	1	1	1	6
Sperling et al. (2022) [51]	1	0	1	1	1	0	1	1	1	7
Stavem et al. (2021) [52]	1	0	1	1	1	0	1	1	1	7
Sun et al. (2022) [53]	1	0	1	1	1	0	1	1	1	7
Thiele et al. (2022) [54]	1	1	1	1	1	1	1	1	1	9
Tleyjeh et al. (2022) [55]	1	1	1	1	1	1	1	1	0	8
Townsend et al. (2021) [56]	1	1	1	0	1	0	1	1	1	7
Verveen et al. (2022) [58]	1	1	1	1	1	1	1	1	1	9
Wensink et al. (2023) [59]	1	0	1	1	1	0	1	0	0	5
Zhang et al. (2021) [60]	1	1	1	1	1	1	1	1	1	9

**Table 4:** Quality assessment based on AHRQ. Y: Yes; U: Unclear; AHRQ: Agency for Healthcare Research and Quality

Study	Study design	1	2	3	4	5	6	7	8	9	10	11	AHRQ	Quality
Alotibi et al. (2023) [24]	Cross-sectional	Y	Y	U	Y	Y	U	U	U	U	U	U	4	Moderate
Aly et al. (2021) [25]	Cross-sectional	Y	Y	Y	Y	Y	U	U	U	U	U	U	5	Moderate
Delgado-Alonso et al. (2022) [26]	Cross-sectional	Y	U	Y	Y	Y	U	U	U	U	U	U	4	Moderate
El Sayed et al. (2021) [28]	Cross-sectional	Y	Y	Y	Y	Y	Y	U	U	U	U	U	6	Moderate
Grover et al. (2021) [36]	Cross-sectional	Y	Y	U	Y	Y	Y	U	U	U	U	U	5	Moderate
Khatib et al. (2022) [39]	Cross-sectional	Y	Y	Y	Y	Y	Y	Y	U	U	U	U	7	Moderate
Twomey et al. (2022) [57]	Cross-sectional	Y	Y	Y	Y	Y	Y	Y	U	U	Y	U	8	High

Overall Pooled Prevalence

**Figure 2:**
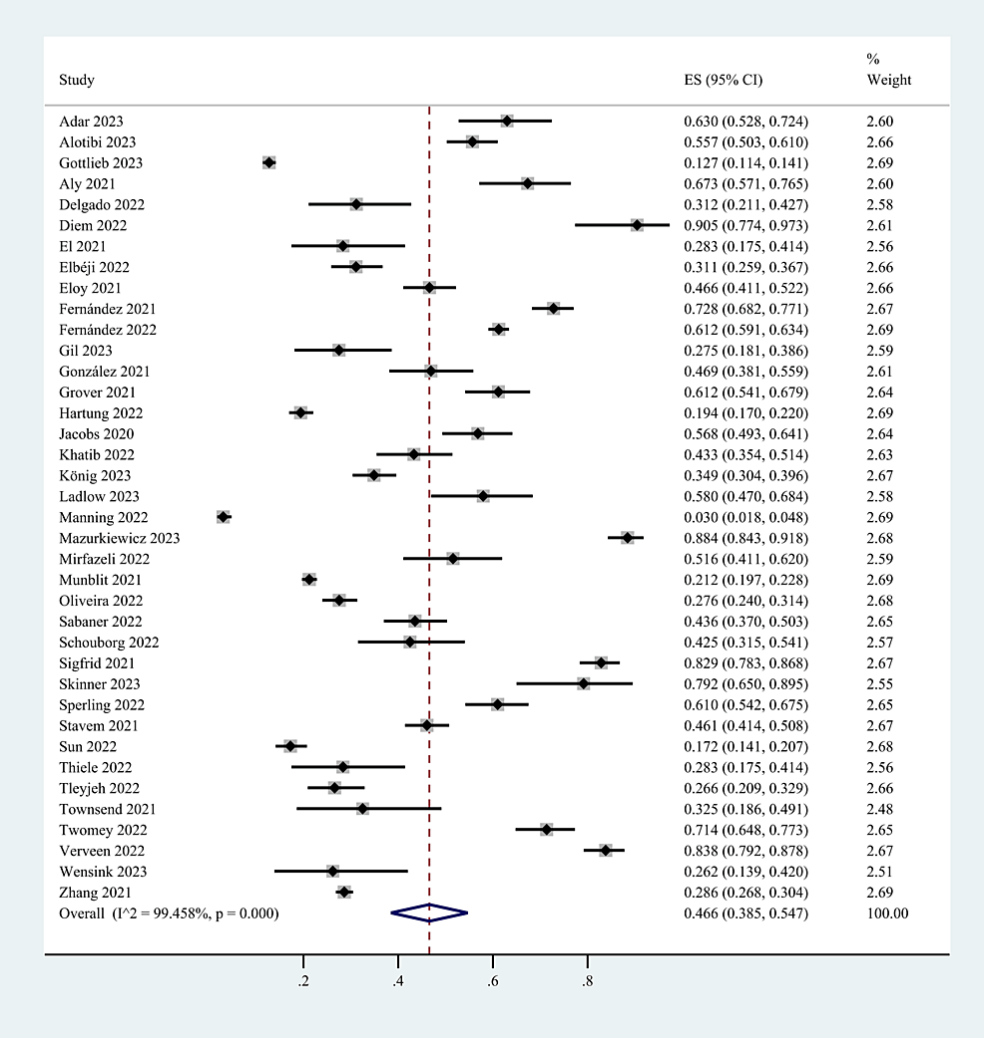
The pooled prevalence of post-COVID-19 fatigue. CI: Confidence interval; ES: Effect size

The overall prevalence of post-COVID-19 fatigue is shown in Figure 2. Because there is a significant heterogeneity (I^2^ = 99.5%) in the analysis results, this study chose the random-effects model for data analysis. The pooled prevalence of post-COVID-19 fatigue was 46.6% (95% CI: 38.5%-54.7%).

Associated Factors of Post-COVID-19 Fatigue

The factors in this meta-analysis included female gender, age, BMI, education, occupation, depression, clinical severity, number of acute COVID symptoms, number of medications, length of hospital stay days, comorbidities, hypertension, lung disease, former smoking, current smoking, and marital status. This study categorized these 17 factors into three categories: demographic characteristics, clinical manifestations and therapy of COVID-19 infection, and basic health status. The extracted factors and statistical results of the meta-analysis are shown in Table 5. Finally, the results revealed that female gender, older age, clinical severity, and the number of acute COVID symptoms, hypertension, lung disease, and depression are risk factors for post-COVID-19 fatigue.

**Table 5:** The meta-analysis results of associated factors of post-COVID-19 fatigue. OR_L_: Lower limit of confidence interval for odds ratio; OR_U_: Upper limit of confidence interval for odds ratio

Variables	Factors	Pooled OR	OR_L_	OR_U_	p-value
Demographic characteristics	Gender	0.40	0.24	0.56	0.000
	Older age	0.04	0.01	0.07	0.027
	BMI	1.02	0.98	1.05	0.321
	Occupation	0.91	0.29	2.90	0.873
	Education	0.59	-0.32	1.50	0.203
	Marital status	1.08	0.44	2.66	0.867
Clinical manifestations and therapy of COVID-19 infection	Clinical severity	0.66	0.24	1.09	0.002
	Number of acute COVID symptoms	3.23	1.83	5.69	0.000
	Number of medications	1.60	0.31	8.25	0.572
	Length of hospital stay days	0.99	0.94	1.04	0.624
Basic health status	Comorbidities	-0.12	-0.53	0.30	0.579
	Hypertension	1.24	1.08	1.42	0.002
	Lung disease	2.71	1.07	6.89	0.036
	High VAS	1.15	0.78	1.68	0.485
	Former smoking	0.36	-0.89	1.61	0.573
	Current smoking	0.08	-0.20	0.36	0.577
	Depression	1.55	1.01	2.39	0.045

Demographic Characteristics

**Figure 3:**
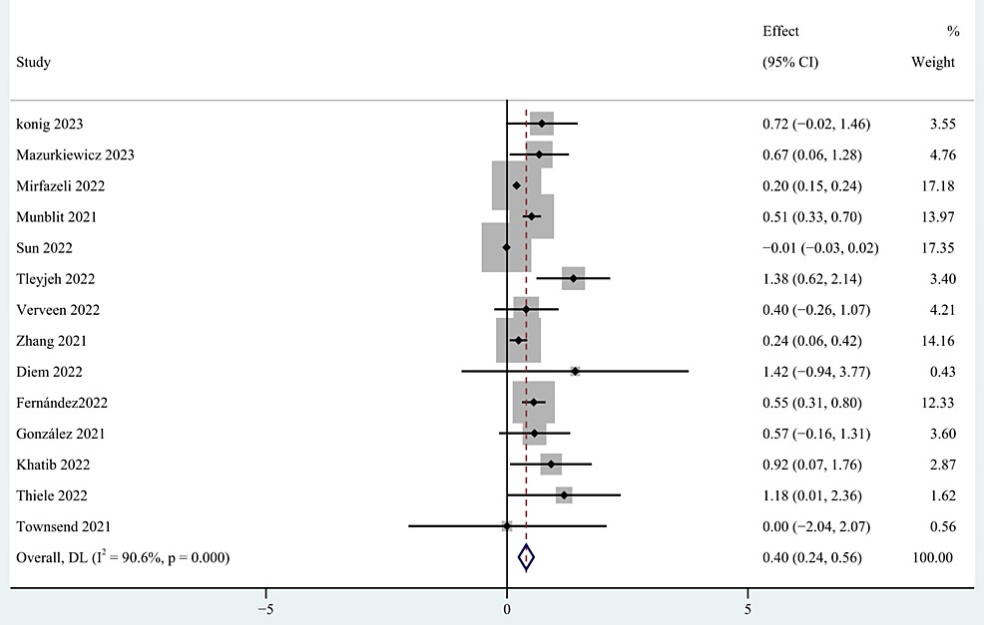
The forest plot of pooled OR between female gender and post-COVID-19 fatigue. OR: Odds ratio

**Figure 4:**
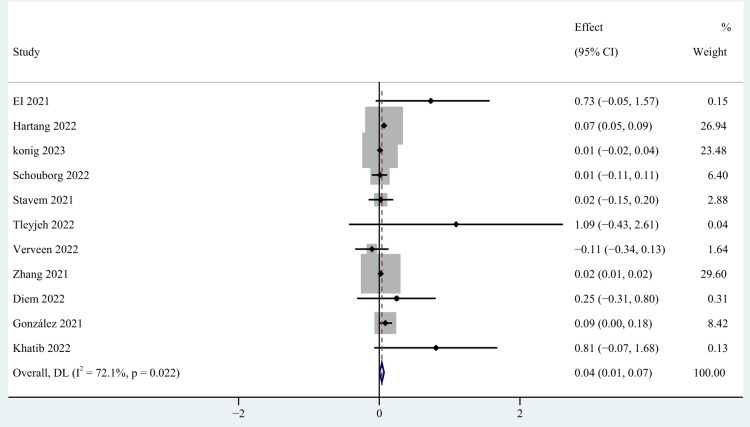
The forest plot of pooled OR between age and post-COVID-19 fatigue. OR: Odds ratio

Gender [27,32,34,39,40,43-45,53-56,58,60], older age [27,28,34,37,39,40,48,52,55,58,60], body mass index (BMI) [27,34,39,40,52,54,56], occupation [39,59], education [40,52], and marital status [39,40,52] were included as factors associated with the demographic characteristics of patients. This study found that gender and older age were risk factors for persistent post-COVID-19 persistent fatigue groups, with a pooled OR of 0.40 (95% CI: 0.24-0.56; p < 0.0001; Figure 3) and 0.04 (95% CI: 0.01-0.07; p=0.027; Figure 4), but we found no statistical significance between BMI, occupation, education, marital status, and post-COVID-19 fatigue, with a pooled OR of 1.02 (95% CI: 0.98-1.05), 0.91 (95% CI: 0.29-2.90), 0.59 (95% CI: -0.32-1.50), and 1.08 (95% CI: 0.44-2.66).

Clinical Manifestations and Therapy of COVID-19 Infection

**Figure 5:**
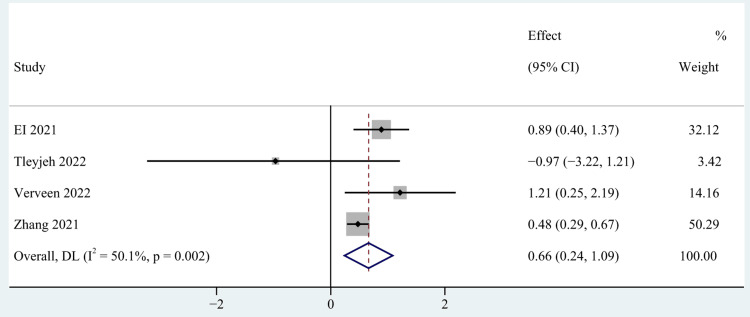
The forest plot of pooled OR between clinical severity and post-COVID-19 fatigue. OR: Odds ratio

**Figure 6:**
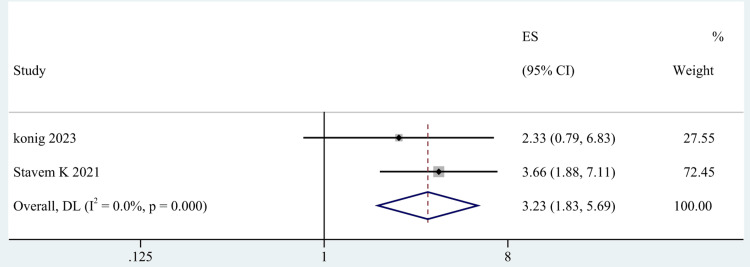
The forest plot of pooled OR between the number of acute COVID symptoms and post-COVID-19 fatigue. OR: Odds ratio

Clinical severity [28,55,58,60], number of acute COVID symptoms [40,52], number of medications [40,56] and length of hospital stay days [53,54,60] were categorized as clinical manifestations and therapy of COVID-19 infection. The meta-analysis based on the random-effects model indicated that clinical severity and the number of acute COVID symptoms were significant risk factors of post-COVID-19 fatigue, with pooled OR of 0.66 (95% CI: 0.24-1.09; p=0.002; Figure 5), and 3.23 (95% CI: 1.83-5.69; p < 0.0001; Figure 6). However, no statistical significance was found between the number of medications and the length of hospital stay days and post-COVID-19 fatigue, with a pooled OR of 1.60 (95% CI: 0.31-8.25) and 0.99 (95% CI: 0.94-1.04) respectively.

Basic Health Status

The comorbidities [34,45,60], preexisting hypertension [34,45,55], lung disease [12,34,51,52,58], high VAS [28,49,52-54,56], former smoking [12,34,48], current smoking [54,60], and depression [34,39,40,52,54,60] were categorized as basic health status. By the random-effects model, the results indicated that preexisting hypertension, lung disease, and depression were risk factors for post-COVID-19 fatigue, with a pooled OR of 1.24 (95% CI: 1.08-1.42; p=0.002), 2.71 (95% CI: 1.07-6.89; p=0.036), and 1.55 (95% CI: 1.01-2.39; p=0.045). However, comorbidities, a high VAS score, former smoking status, and current smoking showed no significant association with post-COVID-19 fatigue, with a pooled OR of -0.12 (95% CI: -0.53-0.30), 1.15 (95% CI: 0.78-1.68), 0.36 (95% CI: -0.89-1.61), 0.08 (95% CI: -0.20-0.36) respectively.

Subgroup Analysis

Based on the evaluation techniques and the continent identified by the WHO regions, subgroup analysis was performed. The subgroup analysis revealed that neither the evaluation methods nor continent was a significant moderator. In terms of the assessment techniques, basic checklists and interviews revealed a lower prevalence of fatigue (41.3%, 95% CI: 25.1-57.4) than did the standardized evaluation techniques (48.7%, 95% CI: 38.6-58.8). The subgroup analysis of the continent revealed that Africa had a greater prevalence of fatigue (51.8%, 95% CI: 44.6-59.0) than America (43.4%, 95% CI: 20.3-66.5), Europe (48.1%, 95% CI: 36.7-59.4), and Asia (43.1%, 95% CI: 32.1-54.0).

Sensitivity Analysis and Publication Bias

**Figure 7:**
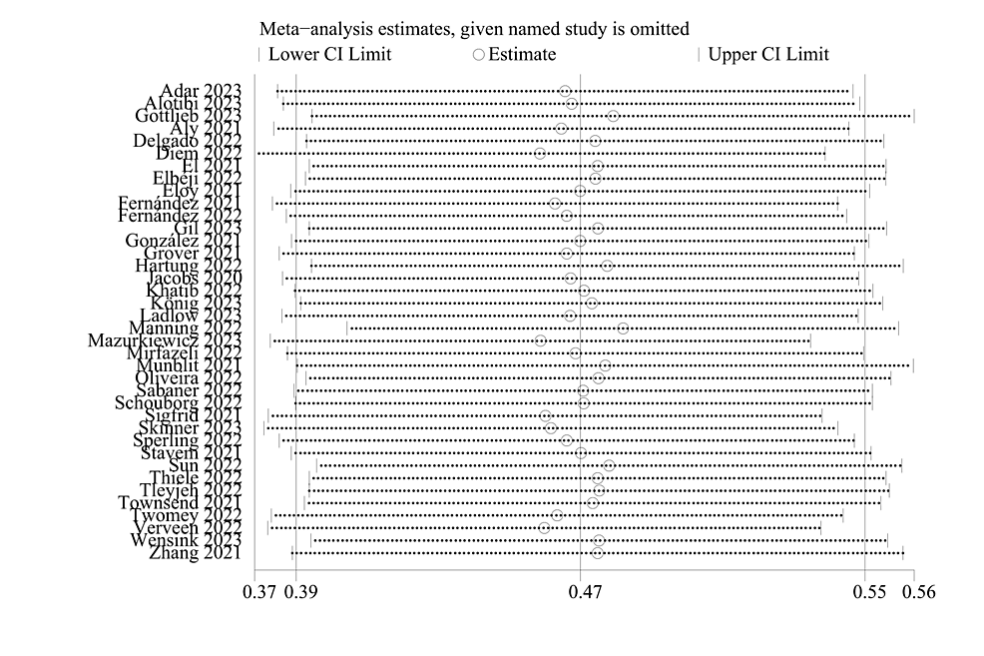
Sensitivity analysis.

**Figure 8:**
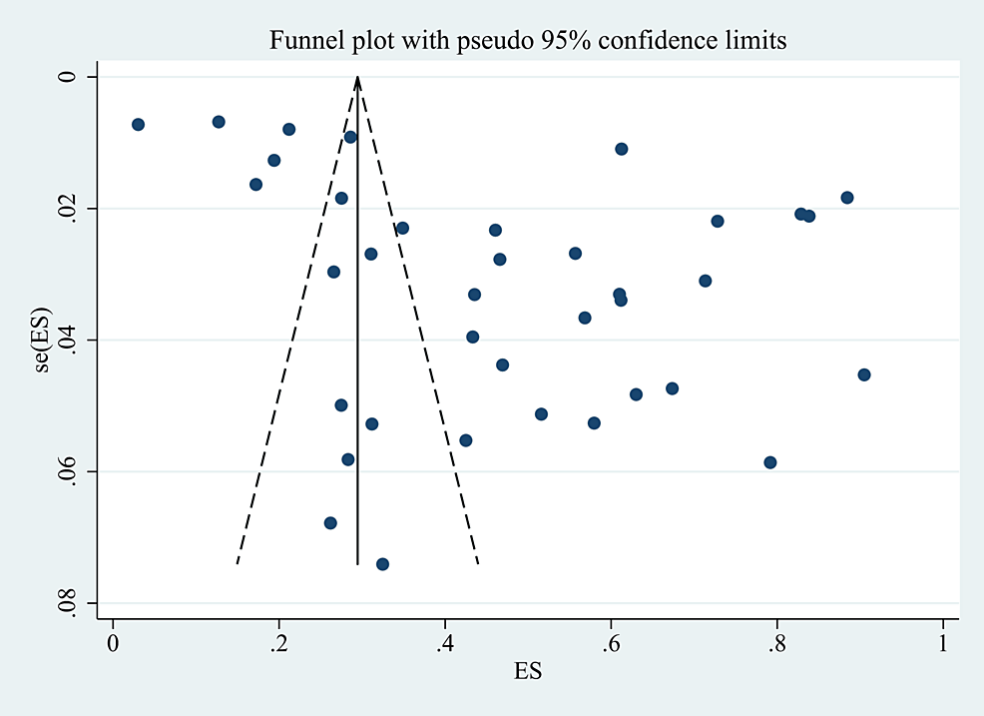
Funnel plot of publication bias.

In addition, we performed a sensitivity analysis and evaluated publication bias using the funnel plot and Egger's test on the included studies, and the sensitivity remained stable after excluding certain studies. Therefore, the sensitivity analysis of this study was considered robust, and the results of the sensitivity analysis and publication bias analysis are shown in Figure 7 and Figure 8.

Discussion

Our study summarized the prevalence and associated factors of post-COVID-19 fatigue, and we extracted available data in 38 studies and 17 associated factors for meta-analysis. Thus, the proportion of people who reported feeling fatigued after COVID-19 was 46.6% (95% CI: 38.5-54.7). This result shows that half of people will experience persistent fatigue after COVID-19 infection, which further emphasizes the need of controlling and treating post-COVID-19 fatigue.

The meta-analysis with a random-effects model showed that female gender, older age, depression, clinical severity, number of acute COVID symptoms, hypertension, and lung disease were significantly associated factors of fatigue. It demonstrates that the occurrence of fatigue is significantly influenced by female gender, and numerous investigations have confirmed this relationship. Several hypotheses may explain this connection, since typical female roles, such as child-rearing, family education and other caregiving responsibilities, remain predominantly female, women are disproportionately affected by measures of enforced segregation and closure [61]. Further research supported the theory that autoimmune mechanisms contributing to the development of Long COVID by X chromosome-linked genes are thought to influence the probability of vulnerability to viral infections and autoimmune illnesses. This could be a potential reason why fatigue is more common in females [62,63].

In addition, older age is also a risk factor for post-COVID-19 fatigue, and the reason may be related to the fact that older age is associated with reduced physical functioning and a diminished ability to recover [64]. Besides, we discovered that post-COVID-19 fatigue is more common in patients who are with depression. Fatigue and depression are both neurological symptoms, and many studies have found there is a potential association between fatigue and depression. A prospective cohort study found patients with depression have higher levels of cytokines systemic inflammation, which can also contribute to post-COVID-19 fatigue [65,66]. Consistent with our findings, prospective research into gender variations in post-COVID-19 syndrome found that women were more likely to experience higher degrees of depression and anxiety as well as lower projected maximal oxygen utilization [67]. Moreover, as depression is exactly the factor that our study found to be associated with post-COVID-19 fatigue, this finding further explains why women are more susceptible to post-COVID-19 fatigue.

Besides, our results showed that fatigue is more likely to happen in patients severely affected by COVID-19 and experiencing more symptoms during their COVID-19 infection. This may be as the immune functions of patients with severe COVID-19 infections are compromised by the virus, leading to long-term tissue damage and greater symptom burden due to pathological inflammation [7,68]. Therefore, proactive therapeutic and preventive measures for patients who have more severe infections and more symptoms of COVID-19 infection are required to minimize the occurrence of post-COVID-19 fatigue after infection and reduce the resultant distress.

Hypertension was shown to be the most prevalent comorbidity in patients with post-COVID-19 fatigue in a cohort analysis of participants experiencing those symptoms. This meta-analysis also showed that patients with hypertension and lung disease had a higher possibility of developing fatigue, while the mechanisms and potential connection between hypertension and lung disease in post-COVID-19 fatigue need to be further explored. However, we can implement preventive and treatment measures to avoid increasing the incidence or severity of fatigue, such as controlling the blood pressure at normal levels and relieving lung infections [69].

Moreover, in the subgroup analysis of continents, we did not find significant heterogeneity differences among continents. For types of evaluation tools, several standardized tools were used for assessing fatigue symptoms, and some studies developed fatigue symptom checklists or questionnaires by themselves. One study validated the use of the Fatigue Severity Scale (FSS) and two single-item screening questions in two groups, and it was reported that the FSS showed a higher internal consistency and construct validity [70]. However, there is no standardized assessment tool or scale for assessing post-COVID-19 fatigue. Therefore, we categorized the fatigue tools used in the included studies as standardized scales or questionnaires and simple checklists or questionnaires. Through subgroup analysis, the results showed that the use of standardized scales or questionnaires could identify more post-COVID-19 fatigue in relation to simple checklists or questionnaires (47.5% vs. 43.2%), and the use of simple checklists or questionnaires might ignore some patients with post-COVID-19 fatigue. Therefore, during assessing fatigue symptoms in patients, it is advisable to choose standardized scales which are more accurate and comprehensive to achieve better accuracy in identifying fatigue symptoms. There is a limited evidence on the optimal assessment tool for post-COVID-19 fatigue, and more clinical studies are necessary to determine the best assessment tool.

While the risk factors for post-COVID-19 syndrome have been extensively studied, fatigue has received less attention. As the most prevalent symptom of post-COVID-19 syndrome, post-COVID-19 fatigue has been shown in numerous research [71]. Therefore, it is necessary to clarify its prevalence and the related factors so that risk prediction models or preventive treatments can be developed for people at high risk of this symptom, and individualized interventions and care can be provided for patients suffering from fatigue. Some studies have tried experiments of different interventions to mitigate the severity of fatigue, and some interventions have achieved positive outcomes. This study demonstrated that post-COVID-19 fatigue was related to the clinical severity of the COVID-19 infection, the number of symptoms, preexisting hypertension, and lung disease [17-19]. Future clinical trials are required to improve awareness of patients with fatigue and related factors, and confirm whether therapies using these artificial factors can prevent post-COVID-19 fatigue.

Limitations

This literature study does have several limitations, though. First off, this study's inclusion of studies published in English language. Second, the heterogeneity in the included studies was high, but we analyzed the heterogeneity source from the continents and the evaluation tools of the studies. And we considered that age might be an important factor influencing fatigue. As some studies did not divide age into groups or the standard of grouping was not uniform which resulted in the lack of the available parameter to use age as a grouping condition for subgroup analysis, so we did not ultimately identify the heterogeneity's underlying sources of heterogeneity. We did not distinguish between physical and mental fatigue due to a lack of sufficient data. There may be differences in the influences related to physical and mental fatigue, but we did not discuss them separately.

## Conclusions

This systematic review and meta-analysis revealed associated factors of post-COVID-19 fatigue, which can work as distinct contributing characteristics to identify individuals who may be fatigued and to implement targeted and all-encompassing interventions to improve post-COVID-19 fatigue prevention and control. We found female gender, older age, depression, clinical severity of COVID-19 infections and the number of acute COVID symptoms, preexisting hypertension, and lung disease are risk factors for post-COVID-19 fatigue. In addition, we suggest the use of standardized scales or questionnaires in the evaluation of post-COVID-19 fatigue to improve the accuracy of the assessment.
